# Exploiting biomechanical degrees of freedom for fast and accurate changes in movement direction: coordination underlying quick bow reversals during continuous cello bowing

**DOI:** 10.3389/fnhum.2013.00157

**Published:** 2013-04-26

**Authors:** Julius Verrel, Steven Pologe, Wayne Manselle, Ulman Lindenberger, Marjorie Woollacott

**Affiliations:** ^1^Max Planck Society, Center for Lifespan Psychology, Max Planck Institute for Human DevelopmentBerlin, Germany; ^2^School of Music, University of OregonEugene, OR, USA; ^3^Department of Human Physiology and Institute of Neurosciences, University of OregonEugene, OR, USA

**Keywords:** coordination, timing, degrees of freedom, expertise, cello bowing, music performance, movement reversal

## Abstract

Theoretical and empirical evidence suggests that accurate and efficient motor performance may be achieved by task-specific exploitation of biomechanical degrees of freedom. We investigate coordination of the right arm in a task requiring a sudden yet precisely controlled reversal of movement direction: bow reversals during continuous (“legato”) tone production on a stringed instrument. Ten advanced or professional cello players (at least 10 years of practice) and ten age-matched novice players took part in the study. Kinematic data from the bow and the right arm were analyzed in terms of velocity and acceleration profiles, as well as temporal coordination along the arm. As expected, experts' bow velocity and acceleration profiles differed markedly from those of novice participants, with higher peak accelerations and quicker direction changes. Importantly, experts achieved the change in movement direction with a single acceleration peak while novices tended to use multiple smaller acceleration peaks. Experts moreover showed a proximal-distal gradient in timing and amplitudes of acceleration peaks, with earlier and lower-amplitude reversals at more proximal joints. We suggest that this coordination pattern allows generating high accelerations at the end effector while reducing the required joint torques at the proximal joints. This may underlie experts' ability to produce fast bow reversals efficiently and with high spatiotemporal accuracy. The findings are discussed in terms of motor control theory as well as potential implications for musicians' performance and health.

## Introduction

Stringed instrument bowing is a complex coordinative sensorimotor ability acquired through years of deliberate practice (Ericsson et al., 1993). Learning to play a stringed instrument requires the development of new sensorimotor skills, which may differ significantly from those used in everyday activities. For instance, experienced cello players do not show the same trade-off between movement distance, speed and end point accuracy during shifting movements (Chen et al., 2006) as typically found in non-trained individuals (Fitts, 1954; Schmidt et al., 1979). This makes string instrument bow technique an excellent model for studying general questions about motor coordination and its development (Bernstein, 1967, 1996).

The present study addresses a specific aspect of skilled string instrument bowing, namely the question, how cello players achieve the quick reversal of bow direction during prolonged legato (continuous-tone) bowing. While it is physically impossible to achieve a perfect legato, that is, making intermediate changes in bow direction “inaudible,” expert musicians seem to be able to approximate it to a great extent by maintaining the velocity of the bow till the very end of the movement and reversing the movement in a fast, impulse-like manner (Mantel, 1995). A central question concerns how skill-specific velocity and acceleration profiles are achieved by coordination among the joints of the right (bowing) arm. Texts on cello technique emphasize the flexible control of the joints of the arm in bowing. This suggests, that differentiated use of the right arm's degrees of freedom (DOF), as suggested by Bernstein's theory of skill acquisition (Bernstein, 1967) may be at work when experts change bow direction. Yet, how the DOF of the bowing arm are actually coordinated and how this coordinative skill is acquired is not understood.

Thus, the present study aims at characterizing expert cellists' performance of the fast bow reversal required during continuous legato bowing. To this end, we analyzed kinematics of bow movements (velocity and acceleration) and the right arm (joint angles and spatial acceleration profiles) at the times of bow reversal in advanced cellists and age-matched cello novices. We predicted that expert cellists would show faster bow reversals (quantified by acceleration amplitudes and duration of direction change) and more consistent timing of bow acceleration profiles, when compared to cello novices. Regarding the coordination of the right arm, we hypothesized that experts would show greater independence of DOF, as characterized by distinct timing and amplitudes of acceleration profiles along the kinematic chain.

## Materials and methods

The data used for the present study were acquired from the same participants and during the same experimental session as those used in an earlier study (Verrel et al., 2013). However, while the earlier study analyzed the use of DOF during performance of whole-bow movements (i.e., relatively large transport movements), the present study addresses coordination of DOF at bow reversals during shorter bow movements with an emphasis on continuous-tone production.

### Participants

Ten advanced or professional players (3 female, age ± SD: 22.9 ± 4.3 years, age range: 19–32 years) and ten novice players (3 female, 23.5 ± 3.5 years, age range 21–32 years) took part in the study. Advanced or professional players (“experts”) had at least 5 years of cello education (12.4 ± 5.5 years, range: 5–20 years), at least 10 years of total cello experience (14.4 ± 5.1 years, range: 10–24 years), and were students of cello at a conservatory or advanced amateurs. Novices had no prior experience with the cello or any other bowed string instrument. The experiment was approved by local ethics committees (Max Planck Institute for Human Development, Berlin, and University of Oregon, Eugene, OR) and conducted with participants' written informed consent and in accordance with the Declaration of Helsinki.

### Experimental procedure

At the beginning of the experiment, novices received a standardized introduction to cello bowing, approved by a highly experienced cello teacher (one of the authors, SP). This included instructions of how to hold the bow with the right hand, controlling the position and movement of the bow relative to the string, and controlling bowing velocity. In particular, novice participants were explicitly instructed to maintain an orthogonal angle and constant contact point between bow and string, and to move the bow at a constant velocity. For participants without any prior musical experience, additional instructions and practice were given regarding timing their own movements with the metronome. Special care was taken to prevent fatigue or injury in carrying out the unfamiliar movement, by suggesting trying to perform the movements with as little effort as possible, providing breaks, and asking participants to perform relaxing hand and arm movements between the trials.

Due to organizational constraints, the experiment had to be split between two labs (Berlin and Eugene, see author affiliations of Julius Verrel and Marjorie Woollacott). One of the authors (Marjorie Woollacott) was present during the experiments in both labs, ensuring consistency of experimental procedures. Of the 20 participants, eight were tested in Berlin (6 novices, 2 experts) and twelve in Eugene (4 novices, 8 experts). Unfortunately, this means that Group (expert, novice) and Lab (Berlin, Eugene) are confounded variables. We dealt with this methodological issue conservatively, by assessing effects of Group (experts, novices) only after accounting for effects of Lab (see section Statistical Analysis). Kinematic data were acquired using 3D motion capture systems (Berlin: Vicon MX, Oxford, UK, sampling rate 120 Hz; Eugene: Motion Analysis, PEAK Performance Technologies, Englewood, CO, sampling rate 60 Hz). The data acquired in Berlin were down-sampled to 60 Hz during preprocessing.

**Figure 1:**
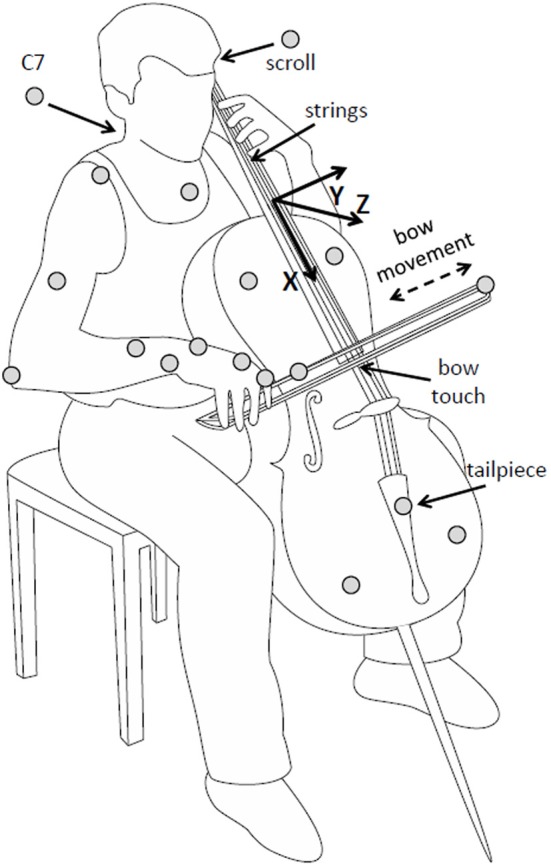
**Illustration of setup, instrument, and task.** Marker positions are indicated as gray circles. The cello-centered coordinate system is indicated (note that the actual origin was defined to be at the scroll marker). Reproduced with permission from Verrel et al. (2013).

Participants wore sleeveless shirts so that the shoulders were free for marker placement. Passive reflective markers (diameter 12 mm) were attached directly on the skin of participants on the trunk (sternum, C7), right arm (acromion, lateral epicondyle of the elbow, lower arm, and wrist), and right hand (first metacarpophalangeal joint and first proximal interphalangeal joint). Cello and bow motion were recorded with additional markers on the cello (scroll and tail piece, defining the “string axis,” and on the cello body, defining the lateral axis) and on the bow (on the tip, and about two-thirds of the way between tip and frog), see Figure 1.

The task consisted of repeated bowing movements on the open A-string (the string with the highest pitch, without using the left hand to change the pitch) at a metronome-prescribed tempo of 80 bows per minute, that is, 40 down-bows and 40 up-bows per minute. Participants were instructed to try producing a continuous tone, emphasizing smooth and fast transitions between up-bows and down-bows. Twenty bowing cycles (up- and down- movements) were acquired per participant.

### Data analysis

Kinematic position data were low-pass-filtered with a bidirectional fifth-order Butterworth filter with a cut-off frequency of 20 Hz. Details of the initial data processing have been reported previously (Verrel et al., 2013). Virtual markers corresponding to estimated joint centers were defined based on individual anthropometric measures. Movement data were transformed to a cello-centered coordinate system, in order to analyze the movement of the bow relative to the string. Joint angles during movement were computed based on an upper-limb model (Rab et al., 2002). Data inspection and information from the cello teaching literature indicated that the most relevant joint angles were: shoulder adduction/abduction, elbow flexion/extension, and wrist and finger flexion/extension. For these angles, larger (positive) values correspond to more abducted (shoulder) and more flexed (elbow, wrist, finger) postures.

**Figure 2:**
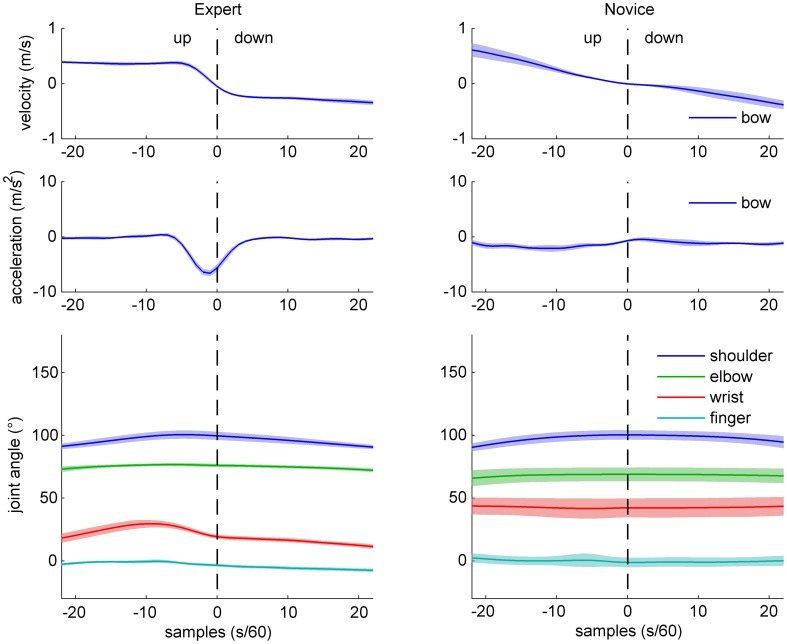
**Sample data of bow kinematics (velocity and acceleration) and joint angle movement for an expert (left) and a novice (right) cello player, during an up-down bow reversal.** Shaded areas indicate SD across movements.

The velocity vector of the bow tip was computed by three-point differentiation. *Bowing velocity* was defined as the component of this velocity vector parallel to the current bow orientation (line through the two bow markers), by computing the inner product of the two vectors. This definition is based on the fact that only movement along the bow's longitudinal axis leads to significant linear movement of the bow on the string, which produces the sound. *Bow acceleration* was defined by three-point differentiation of bow velocity. Zero-crossings of bow velocity were used to identify bow movement *reversals* (changes in direction from up-bow to down-bow, see Figure 2).

Only movements between detected bow reversals with a duration between 0.5 s and 1 s (instructed duration: 0.75 s) were scored as bowing movements. Of these, only those without missing values (for bow position or joint angles) were considered (91.7% of all bowing movements). Only bow reversals between such valid bow movements were analyzed. In order to minimize potential effects of different number of valid bowing movements between participants (and groups), only the first 10 up-down and down-up reversals without missing values were used from each participant.

Acceleration profiles of elbow, wrist, and finger joint center as well as the proximal bow marker were computed by three-point differentiating the position data and subsequently computing the Euclidean vector norm. In order to compare reversal patterns along the kinematic chain (from right shoulder to bow), acceleration data were normalized relative to the right shoulder (i.e., divided by the distance between the respective marker and right shoulder joint center).

For the analysis of bow reversals and the underlying spatiotemporal coordination of the arm, kinematic data were time-locked to bow-reversal events (time window: ±375 ms), separately for up-down and down-up reversals. Within these time windows, the following dependent variables concerning bow kinematics were extracted: *time of peak acceleration* (mean and SD) and *amplitude of peak acceleration, acceleration amplitude at the time of bow reversal*, and *reversal duration* (time taken to switch between ±10% or ±50% of peak velocity). Moreover, the *number of relative acceleration peaks* was defined within each bow reversal interval as the number of local acceleration maxima greater than 50% of the global maximum, with the additional requirement that local maxima had to be separated by at least 100 ms.

In order to characterize coordination of the right arm during bow reversals, several spatial and temporal measures were computed. The *time of peak acceleration* and the *amplitude of acceleration at the time of bow reversal* (normalized relative to the shoulder joint) were computed for the markers on the right arm and the proximal bow marker. Moreover, the *time of joint reversal* was defined as the time at which the corresponding joint angle changed from flexion to extension (abduction to adduction for the shoulder) during up-down bow reversals, and vice versa for down-up reversals.

### Statistical analysis

Statistical comparisons were performed in R (R Development Core Team, 2008; Lawrence, 2011). To control for the confounding effect of Lab (Berlin, Eugene), statistical effects of Group and Bow Direction were analyzed *after* removing any main effects (mean differences) of Lab from the dependent variables. Subsequently, dependent variables were submitted to Two-Way repeated measures ANOVAs with Group (expert, novice) as between-subject factor and Direction (up-down, down-up) as within-subject factor. Interaction effects were scrutinized by pairwise *t*-tests.

Spatiotemporal coordination of the right arm was further analyzed using ANOVAs with Group as between-subject factor, and Direction (up-down, down-up) and Marker (elbow, wrist, hand, and bow), or Joint (shoulder, elbow, wrist, and finger), as within-subjects factors. The effects of Direction and Marker were also analyzed separately for each group by means of ANOVAs with Direction (up-down, down-up) and Marker or Joint as within-subject factors. Marker and Joint were defined as ordered factors with a proximal-to-distal ordering. Significant interaction effects were scrutinized by partial ANOVAs and pairwise *t*-tests. For the relative temporal measures, one-sample *t*-tests were used to test whether the dependent variable systematically differed from zero (i.e., the time of bow reversal).

Statistical analyses were performed with a significance threshold of 0.05. *Post-hoc* analyses (pairwise *t*-tests) were corrected for multiple comparisons (Holm, 1979).

## Results

### Sample data

Sample data for bow velocity, bow acceleration, and joint angles of the right arm are shown in Figures 2 (up-down reversals) and 3 (down-up reversals) for one expert and one novice participant. Marked differences are evident between the two participants with respect to bow kinematics, with the expert showing a relatively flat velocity profile before and after the reversal, and a very succinct acceleration peak just before the time of bow reversal. In contrast, the novice's velocity pattern appears more bell-shaped (due to time-locking to bow reversal, only half of the bell-shape is visible), and a more distributed acceleration pattern, possibly with multiple peaks. Comparison of joint angle patterns suggests smaller variability in the expert compared to the novice, and—for the up-down reversal—a distinct movement at the wrist angle anticipating the bow reversal.

**Figure 3:**
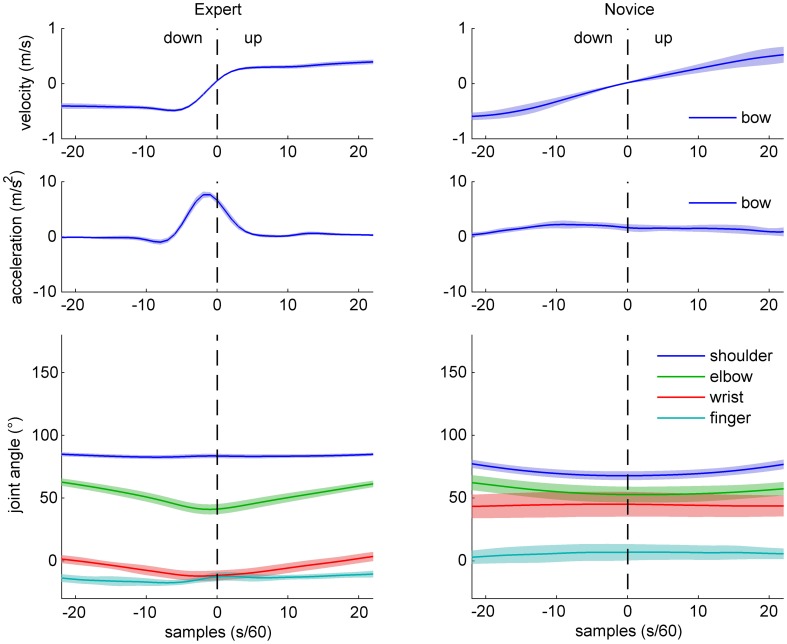
**Sample data of bow kinematics (velocity and acceleration) and joint angle movement for an expert (left) and a novice (right) cello player, during a down-up bow reversal.** Shaded areas indicate SD across movements.

**Figure 4:**
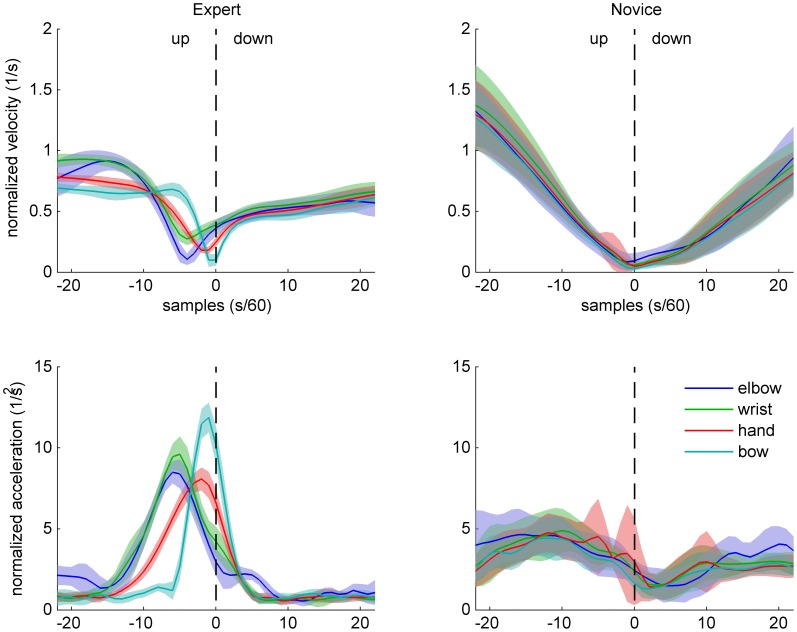
**Sample data of arm and bow kinematics (velocity and acceleration, normalized relative to the shoulder joint) for an expert (left) and a novice (right) cello player, during an up-down bow reversal.** Shaded areas indicate SD across movements.

Normalized velocity and acceleration patterns of the same two participants are shown in Figures 4 (up-down) and 5 (down-up). The expert's movement patterns show low spatiotemporal variability within and clear separation of timing and/or amplitude between the markers. In contrast, the novice's movements show great variability and no clear separation between the markers.

**Figure 5:**
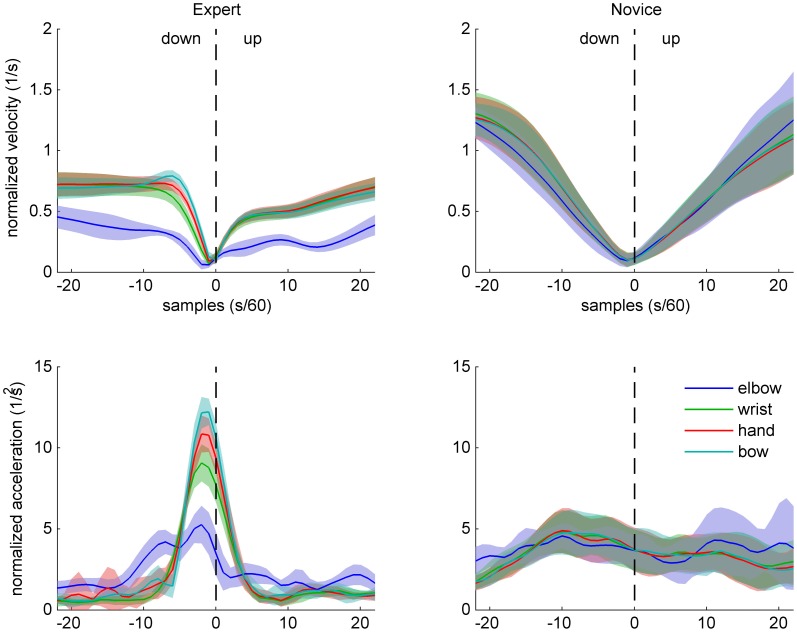
**Sample data of arm and bow kinematics (velocity and acceleration, normalized relative to the shoulder joint) for an expert (left) and a novice (right) cello player, during a down-up bow reversal.** Shaded areas indicate SD across movements.

These observations are in line with the prediction that experts' bowing movements are characterized by skill-specific kinematic patterns (different from bell-shaped profiles typically found in point-to-point movements, e.g., Morasso, 1981) at the bow level and differentiated coordination of the right arm.

### Bow kinematics

**Figure 6:**
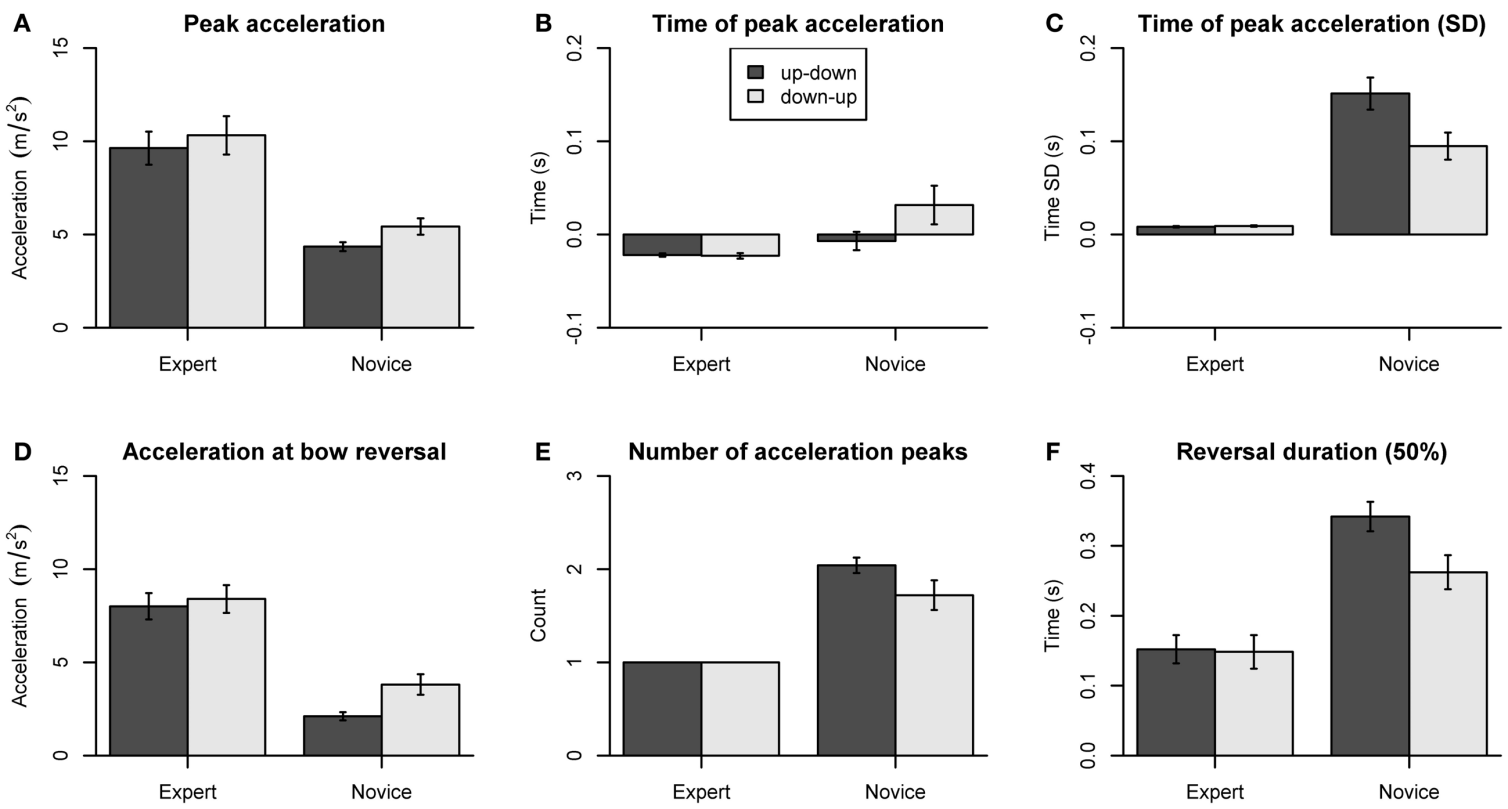
**Dependent variables derived from bow kinematics. (A)** Peak acceleration. **(B)** Time of peak acceleration. **(C)** Variability of time of peak acceleration (SD). **(D)** Acceleration at bow reversal. **(E)** Number of acceleration peaks. **(F)** Reversal duration (±50% of peak velocity). Error bars indicate SE across participants.

The dependent variables concerning bow kinematics are summarized in Figure 6. Peak acceleration amplitudes showed a main effect of Group [*F*_(1, 18)_ = 16.70, *p* < 0.001] and Direction [*F*_(1, 18)_ = 11.61, *p* = 0.003]. Acceleration amplitudes (Figure 6A) were larger in experts compared to novices (*p* < 0.01 for both directions), and larger for down-up than for up-down reversals in novices (*p* = 0.01). The time of peak acceleration (Figure 6B) was consistently negative in experts (*p* < 0.001 for both directions) but showed no systematic deviation from zero in novices. The ANOVA showed a main effect of Group [*F*_(1, 18)_ = 4.46, *p* = 0.025], indicating earlier time of peak acceleration in experts compared to novices. The variability (SD) of time of peak acceleration (Figure 6C) showed main effects of Group [*F*_(1, 18)_ = 31.93, *p* < 0.001], Direction [*F*_(1, 18)_ = 15.63, *p* < 0.001], and a Group by Direction interaction [*F*_(1, 18)_ = 16.50, *p* < 0.001]. *Post-hoc* comparisons showed that variability was smaller in experts than in novices (*p* < 0.01, both directions) and greater for up-down than for down-up reversals in novices (*p* < 0.01).

The acceleration at bow reversal (Figure 6D) showed main effects of Group [*F*_(1, 18)_ = 22.74, *p* < 0.001], Direction [*F*_(1, 18)_ = 13.32, *p* = 0.002], and a Group by Direction interaction [*F*_(1, 18)_ = 5.21, *p* = 0.035]. *Post-hoc* comparisons showed the acceleration amplitude was larger in experts than in novices (*p* < 0.01 for both directions) and larger for down-up than for up-down reversals in novices (*p* = 0.008). The number of acceleration peaks (Figure 6E) was larger in novices than in experts, as shown by a main effect of Group [*F*_(1, 18)_ = 36.58, *p* < 0.001]. The reversal duration, that is, the time taken to switch from −50% to +50% of peak velocity (Figure 6F) showed main effects of Group [*F*_(1, 18)_ = 13.46, *p* = 0.002], Direction [*F*_(1, 18)_ = 33.13, *p* < 0.001], and a Group by Direction interaction [*F*_(1, 18)_ = 27.43, *p* = 0.001]. The reversal duration was shorter in experts than in novices (up-down: *p* < 0.001, down-up: *p* = 0.063), and longer for up-down than for down-up reversals in novices (*p* < 0.001). The reversal duration for switching between ±10% of peak velocity (not shown in the figure) showed the same statistical pattern, with main effects of Group [*F*_(1, 18)_ = 26.34, *p* < 0.001], Direction [*F*_(1, 18)_ = 17.51, *p* < 0.001], and a Group by Direction interaction [*F*_(1, 18)_ = 17.66, *p* < 0.001].

Thus, as expected, experts produced faster and more precisely timed bow reversals than novices. Moreover, experts appear to be able to reverse bow direction with a single acceleration peak. In contrast, novices often showed multiple acceleration peaks. Finally, novices but not experts showed differences between up-down and down-up reversals for some of the dependent measures. This indicates that experts are able to stabilize bow kinematics across different body configurations and that this ability is non-trivial.

### Arm coordination

**Figure 7:**
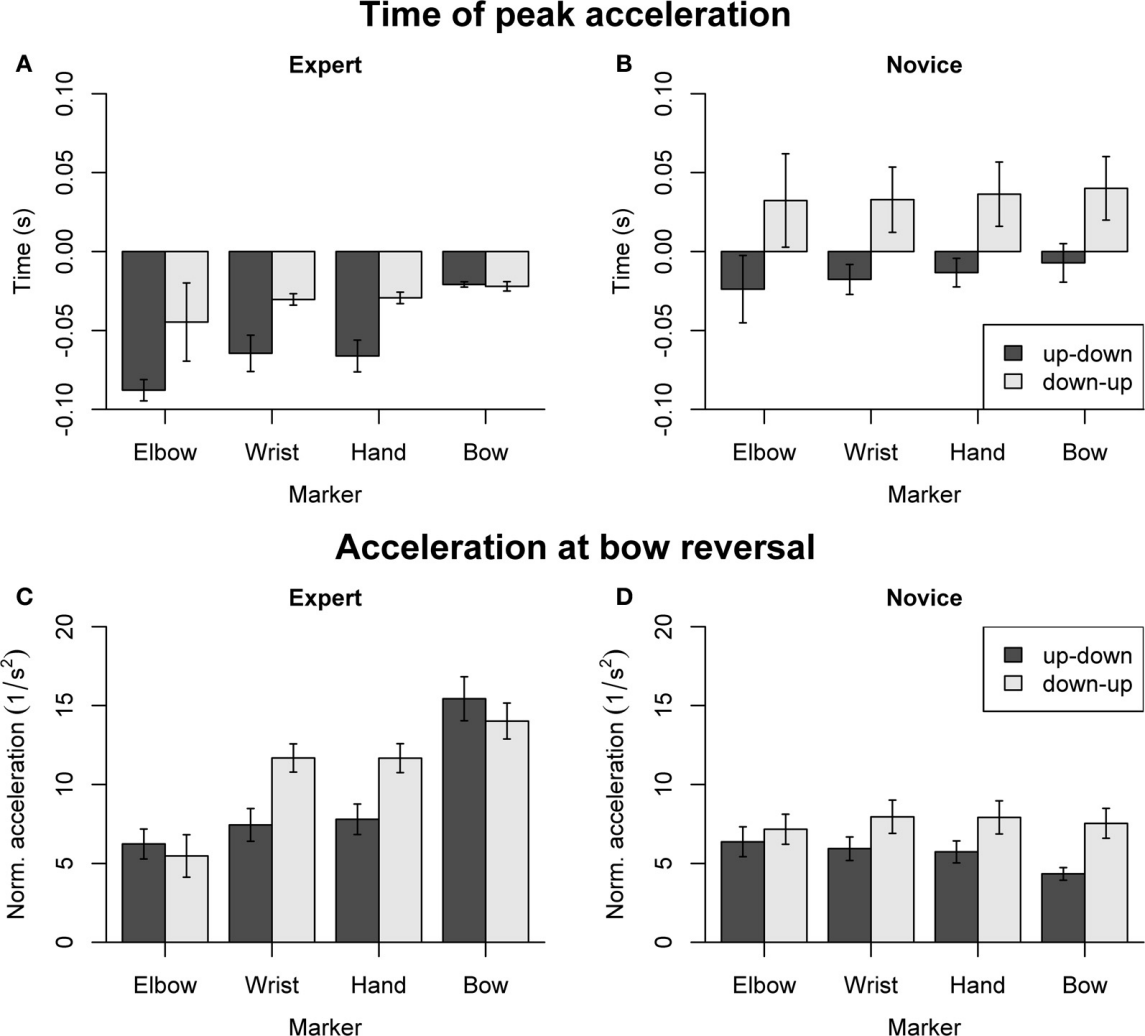
**Dependent variables derived from acceleration patterns of the bow and right arm, normalized relative to the right shoulder. (A,B)** Time of peak acceleration. **(C,D)** Acceleration at bow reversal. Error bars indicate SE across participants.

Dependent variables concerning arm coordination are summarized in Figure 7, separately for experts (Figures 7A,C) and novices (Figures 7B,D). For the time of peak acceleration (Figures 7A,B), the omnibus ANOVA (with factors Group, Direction, and Marker) showed main effects of Group [*F*_(1, 18)_ = 13.27, *p* = 0.002], Direction [*F*_(1, 18)_ = 8.46, *p* = 0.009], and Marker [*F*_(3, 54)_ = 5.91, *p* = 0.001]. In experts (Figure 7A), main effects of Direction [*F*_(1, 9)_ = 13.72, *p* = 0.005] and Marker [*F*_(3, 27)_ = 9.74, *p* < 0.001] were found. Marker showed a significant linear trend [*F*_(1, 27)_ = 25.81, *p* < 0.001] and no higher-level trends, indicating that the time of peak acceleration showed a systematic proximal-to-distal progression. Pairwise comparisons confirmed that the peak acceleration occurred later at the bow than at the hand (*p* = 0.003), wrist (*p* = 0.003), and elbow marker (*p* < 0.001) during up-down reversals. No significant effects of Direction or Marker were found in novices (Figure 7B). Separate ANOVAs (Group, Direction) for each arm marker showed that acceleration peaks occurred earlier in experts than in novices for all Markers: comparable patterns of main effects were found for the hand [Group: *F*_(1, 18)_ = 11.86, *p* = 0.003, Direction: *F*_(1, 18)_ = 12.39, *p* = 0.002], wrist [Group: *F*_(1, 18)_ = 9.21, *p* = 0.007, Direction: *F*_(1, 18)_ = 11.45, *p* = 0.003] and elbow marker [Group: *F*_(1, 18)_ = 8.85, *p* = 0.008, Direction: *F*_(1, 18)_ = 4.03, *p* = 0.060].

For the acceleration at bow reversal (Figures 7C,D), the omnibus ANOVA showed main effects of Group [*F*_(1, 18)_ = 6.36, *p* = 0.021], Direction [*F*_(1, 18)_ = 11.33, *p* = 0.034], Marker [*F*_(3, 54)_ = 22.77, *p* < 0.001], as well as interactions of Group and Marker [*F*_(3, 54)_ = 34.11, *p* < 0.001], Direction and Marker [*F*_(1, 18)_ = 9.41, *p* < 0.001], and Group, Direction and Marker [*F*_(3, 54)_ = 9.78, *p* < 0.001]. The separate ANOVA for experts (Figure 7C) showed a main effect of Marker [*F*_(3, 27)_ = 31.92, *p* < 0.001] and a Direction by Marker interaction [*F*_(3, 27)_ = 10.12, *p* < 0.001]. Marker showed significant linear [*F*_(1, 27)_ = 86.38, *p* < 0.001] and cubic trends [*F*_(1, 27)_ = 8.38, *p* = 0.007], indicating that acceleration amplitude increased from proximal to distal markers. Pairwise comparisons showed that, during up-down reversals, the acceleration was greater at the bow than at the elbow, wrist and hand (all *p* < 0.001). During down-up reversals, the acceleration was smaller at the elbow compared to wrist, hand and bow (all *p* < 0.002). The separate ANOVA for novices (Figure 7D) showed main effects of Direction [*F*_(1, 9)_ = 8.58, *p* = 0.017] and Marker [*F*_(3, 27)_ = 3.62, *p* = 0.026], and a Direction by Marker interaction [*F*_(3, 27)_ = 6.54, *p* = 0.002]. However, *post-hoc* comparisons did not reveal any significant pairwise differences. Separate ANOVAs for each Marker did not show main effects of Group, but main effects of Direction for the hand and wrist marker [hand: *F*_(1, 18)_ = 20.08, *p* < 0.001, wrist: *F*_(1, 18)_ = 20.81, *p* < 0.001], with larger acceleration for down-up compared to up-down reversals.

**Figure 8:**
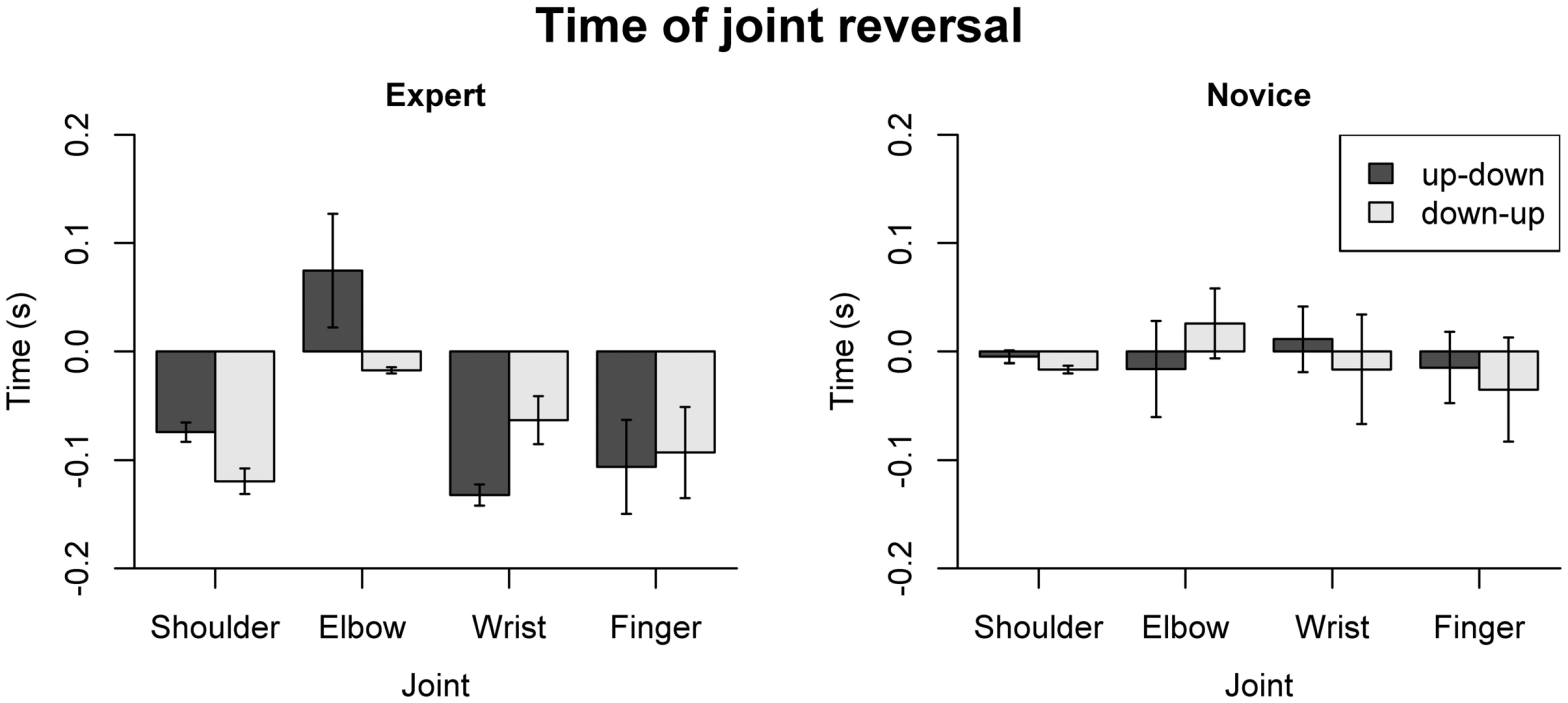
**Dependent variables describing the timing of joint reversals.** Error bars indicate SE across participants.

For the timing of joint angle reversals (Figure 8), the omnibus ANOVA showed main effects of Group [*F*_(1, 18)_ = 7.02, *p* = 0.16] and Joint [*F*_(3, 54)_ = 4.17, *p* = 0.01], and a three-way interaction of Group, Direction, and Joint [*F*_(3, 54)_ = 3.42, *p* = 0.024]. For experts, a main effect of Joint [*F*_(3, 27)_ = 10.31, *p* < 0.001] and a Joint by Direction interaction [*F*_(3, 27)_ = 3.55, *p* = 0.028] were found. Pairwise comparisons showed significant differences between shoulder and elbow (*p* = 0.018), elbow and wrist (*p* < 0.001) and elbow and finger joint (*p* = 0.004) for the up-down reversal, and significant differences between shoulder and elbow joint (*p* = 0.033) for down-up reversals. No significant effects of Joint or Direction were found for the novices. Separate analyses for each joint showed, for the wrist joint, a main effect of Group [*F*_(1, 18)_ = 4.57, *p* = 0.046] and a Group by Direction interaction [*F*_(1, 18)_ = 9.34, *p* = 0.007], with earlier joint reversal in the experts compared to novices during up-down reversals; and for the shoulder joint, main effects of Group [*F*_(1, 18)_ = 36.53, *p* < 0.001], Direction [*F*_(1, 18)_ = 17.89, *p* < 0.001], and a Group by Direction interaction [*F*_(1, 18)_ = 6.24, *p* = 0.024]. *Post-hoc* comparisons for the shoulder joint showed that experts had earlier joint reversals than novices (*p* < 0.005 for both directions), and that in experts, down-up reversals occurred earlier than up-down reversals (*p* = 0.008). One-sample *t*-tests showed that joint reversals of the experts occurred prior to bow reversal, for the wrist joint (up-down: *p* < 0.001, down-up: *p* = 0.057) and shoulder joint (*p* < 0.001 for both directions). For the novices, this was only the case for the shoulder joint and only during down-up reversals (*p* = 0.002). For the experts, the reversal at the elbow joint during up-down reversals occurred *after* bow reversal (*p* < 0.001).

Summing up, the analysis of acceleration patterns along the right arm reveals a proximal-to-distal gradient in experts, which is not present in novices. Experts exhibited an anticipatory coordination pattern, leading to earlier peak accelerations and smaller accelerations at the time of bow reversal at more proximal body parts. No such pattern was visible in the novices. Moreover, experts showed earlier reversals at the wrist and shoulder joint, which moreover preceded bow reversal. In novices, such a pattern was only present (and to a lesser extent) for the shoulder joint during down-up reversals.

## Discussion

We analyzed the kinematics of bowing movements and underlying arm coordination. Regarding bow kinematics, our results confirm the prediction that expert cellists show quicker and more precisely timed bow reversals with greater acceleration amplitudes than cello novices. The analysis of the kinematics of the right (bowing) arm showed that experts used differentiated coordination patterns, with proximal-to-distal gradients in the timing and amplitudes of acceleration peaks along the kinematic chain, as well as characteristic anticipatory timing of joint angle reversals relative to the bow reversal. In contrast, novices tended to move the entire arm as one unit, with no systematic proximal-distal or anticipatory coordination pattern. The findings are discussed in more detail below.

### Bow kinematics

Point-to-point arm movements typically show uniform “bell-shaped” velocity profiles (Morasso, 1981), which have been explained as the motor system satisfying certain optimality criteria, such as movement smoothness (Flash and Hogan, 1985; Todorov and Jordan, 1998). Cellists also make repeated arm movements between specified positions when bowing. Importantly, however, the goal of cello bowing movements is not to reach a particular spatial target, but to produce a tone of specific duration and acoustic characteristics. During continuous-tone (“legato”) bowing, minimizing tone variability may require keeping the bow velocity uniform *during* the bow movement and quickly inverting bow velocity at the time of bow *reversal*. Thus, kinematic profiles of bow movements are expected to differ substantially from normal arm movements in skilled cellists.

Experts performed the bow reversal in a very short time, switching between ±50% of peak velocity in about 150 ms on average, while novices took about twice as long to switch direction. Experts' fast bow reversals were characterized by large and consistently timed acceleration peaks. Importantly, experts achieved bow reversal with a single acceleration peak, serving both to decelerate the preceding movement and to accelerate into the subsequent movement. In contrast, novices showed less pronounced and more distributed acceleration patterns, with multiple peaks of deceleration and acceleration. This suggests that the novices in the present study may have resorted to a “default mode” of arm movement kinematics (the bell-shaped profile), with separate acceleration peaks before and after bow reversal. There may also be a perceptual component to this, as untrained observers systematically perceive visual motions that start with an acceleration phase as having “more constant” velocity profiles compared to visual stimuli that *actually* have constant velocity (Runeson, 1974).

Replacing “default” kinematic patterns by more task-adjusted ones likely is an important step in the acquisition of cello bowing in beginning cellists. Studies with cellists of different expertise levels, in particular beginners with longer exposure than the complete novices in the present study, would allow mapping the time course of acquisition of this ability in more detail.

### Coordination

According to Bernstein's theory of skill acquisition (Bernstein, 1967), mastery of complex skills depends on the flexible, differentiated coordination of biomechanical DOF. Bernstein proposed that early phases of motor learning (i.e., lower skill levels) may be characterized by “freezing” of biomechanical DOF (e.g., joint angles), leading to *en bloc* motion of multiple body parts. Such a mode of function might facilitate performance by reducing task complexity, but likely comes at the cost of increased muscle activity (co-contraction) and may prevent the exploitation of inter-segmental dynamics during task performance. In contrast, Bernstein proposed that higher skill levels are characterized by more differentiated use of DOF, exploiting motor equivalence and inter-segmental torques for more efficient and flexible motor performance.

Our analysis of the spatiotemporal coordination of the right arm during bow reversal supports this view. Expert cellists' arm movements were characterized by differentiated timing of acceleration peaks and joint reversals along the kinematic chain of the right arm. Spatial acceleration peaks tended to occur earlier at proximal than at distal parts of the arm. This was in stark contrast to novices' temporal coordination patterns, which did not show systematic differences in timing along the kinematic chain.

As discussed above, continuous-tone bowing requires fast changes in bow direction, which experts (but not novices) were indeed able to achieve. Such quick direction reversals require a high acceleration of the bow, which—if carried out by the entire arm *en bloc*—would involve high torques at the shoulder and elbow. Such high torques would be both energetically costly and a potential injury risk. Indeed, prevalence of musculoskeletal problems is high in string players (Kreutz et al., 2008), including in particular right shoulder injury (Rickert et al., 2012). In contrast, a more differentiated use of the right arm, with different parts changing direction at different times, may allow reducing and distributing the torque required to perform the bow reversal quickly. In line with this, acceleration amplitudes showed a proximal-to-distal gradient in experts, with lower acceleration peaks at more proximal joints. Novices did not show such a gradient, and even a tendency, for up-down reversals, of *decreasing* acceleration amplitudes from proximal to distal parts of the arm.

Thus, the analysis of acceleration amplitudes along the kinematic chain suggests a functional interpretation of the observed differences in timing between experts and novices. That is, the observed sequential organization may underlie experts' ability to quickly and accurately reverse bow direction while minimizing the required acceleration amplitudes of the arm (and hence joint torques). Unfortunately, our setup did not allow for inverse dynamic analyses of bow reversals, which would allow quantitative statements about the torques generated at different joints as a function of the temporal coordination patterns, in particular the exploitation of interaction torques (Hollerbach and Flash, 1982; Hoy and Zernicke, 1986; Schneider et al., 1989), which arise at one body part due to the movement of adjacent body parts.

Besides the timing and amplitudes of spatial acceleration profiles along the right arm, we also analyzed the timing of joint movement reversals. In experts, the wrist and the shoulder joint angle reversal consistently preceded the time of bow reversal. In contrast, novices only showed anticipatory joint reversals for the shoulder angle and only during down-up reversals. An unexpected finding in this context was that the elbow joint reversal in experts reliably occurred *after* the bow reversal during up-down bow reversals. This likely reflects the fact (not further investigated here) that, in down-bow movements, the elbow contributes most to bow transport during the later phase of the bow movement (Mantel, 1995, pp. 168/169), allowing elbow extension to start after bow reversal.

Proximal-to-distal gradients have previously been found for throwing and hitting tasks, in which the velocity of the end effector has to be maximized (Southard, 1989; Putnam, 1991, 1993). For throwing movements, inverse dynamics analyses suggest that interaction torques are exploited in a proximal-to-distal manner to maximize end point velocity (Hirashima et al., 2003, 2008). Proximal-to-distal temporal patterns have also been described for piano keystrokes (Furuya and Kinoshita, 2007), during which end point velocity needs to be precisely controlled but not necessarily maximized. Inverse dynamic analysis of piano key strokes indicated that experts compared to novices generated greater muscle torques at proximal joints, exploiting resulting interaction torques to reduce distal muscle torques (Furuya et al., 2009). Finally, proximal-distal gradients have also been found in a cyclic clay kneading tasks, with experts exhibiting more differentiated phase relationships between body parts (Yamamoto and Fujinami, 2008).

To our knowledge, the present study demonstrates for the first time a proximal-to-distal pattern for a task requiring high and precisely timed *acceleration* profiles of an end effector. Further research, in particular inverse dynamic analyses and simulation studies are needed to scrutinize the functional role of the observed spatiotemporal coordination patterns. Future research should also address the question in which way the axial skeleton is involved in skilled cello bowing, both with respect to bow transport and maintenance of whole-body equilibrium.

An important question concerns how the coordinative skill underlying quick bow reversals is acquired. As proposed above, this could be studied by assessing changes in coordination longitudinally in beginning cello players. In addition, while this was not the subject of this study, one may speculate how the present findings could be applied to support acquisition of this skill. Research on complex motor skill learning suggests that attending to movement outcomes (in this case: the produced sound) is more effective for learning than attending to details of the underlying coordination (Wulf and Shea, 2002). However, this feedback modality might not be easily accessible to early beginners. In this case, providing augmented feedback of the instant bow velocity of the bow might be a promising approach to learn both maintaining constant velocity during bow transport and quickly changing direction at bow reversal. This being said, beginning learners might not sufficiently explore the “problem space” to discover new modes of coordination when solely focusing on movement outcome. Approximating the experts' coordination pattern may be facilitated by drawing participants' attention to the fact that the DOF of the arm can be organized in a differentiated, sequential way, possibly by evoking the mental image of a whip-like movement (Mantel, 1995, p. 32).

### Conclusions

The present study characterized cello experts' performance of bow reversals during continuous-tone bowing. Experts' fast changes in bow direction, requiring high timed acceleration profiles, were associated with differentiated timing and a proximal-to-distal increase of acceleration amplitudes along the right arm, which likely serves to increase physiological efficiency, a crucial element of healthy music practice and performance.

### Conflict of interest statement

The authors declare that the research was conducted in the absence of any commercial or financial relationships that could be construed as a potential conflict of interest.
